# Optimization and Stability of Heat Engines: The Role of Entropy Evolution

**DOI:** 10.3390/e20110865

**Published:** 2018-11-09

**Authors:** Julian Gonzalez-Ayala, Moises Santillán, Maria Jesus Santos, Antonio Calvo Hernández, José Miguel Mateos Roco

**Affiliations:** 1 Departamento de Física Aplicada, Universidad de Salamanca, 37008 Salamanca, Spain; 2 Instituto de Física Fundamental y Matemáticas, Universidad de Salamanca, 37008 Salamanca, Spain; 3 Centro de Investigación y Estudios Avanzados del IPN Unidad Monterrey, Apodaca, NL 66600, Mexico

**Keywords:** heat engine, local stability, maximum power regime, maximum Omega regime, entropy production

## Abstract

Local stability of maximum power and maximum compromise (Omega) operation regimes dynamic evolution for a low-dissipation heat engine is analyzed. The thermodynamic behavior of trajectories to the stationary state, after perturbing the operation regime, display a trade-off between stability, entropy production, efficiency and power output. This allows considering stability and optimization as connected pieces of a single phenomenon. Trajectories inside the basin of attraction display the smallest entropy drops. Additionally, it was found that time constraints, related with irreversible and endoreversible behaviors, influence the thermodynamic evolution of relaxation trajectories. The behavior of the evolution in terms of the symmetries of the model and the applied thermal gradients was analyzed.

## 1. Introduction

The relevance of heat devices optimization and the searching for global properties of energy converters is increasing due to a growing need of energetic requirements; not only for a better use of available energy, but also for maintenance cost, operation life-time, scale related control issues, etc. All these problems involve entropy production ($\dot{\Delta S}$), heat waste, power output (*P*) and efficiency η [1,2]. Along with maximum power and maximum efficiency or minimum entropy production, compromise based figures of merit have been found very valuable in the optimization analysis of heat devices [3,4,5,6,7,8,9,10,11,12,13,14,15,16,17,18,19]. In this framework, the so-called Ω function, defined as $\Omega \equiv P_{gain}-P_{loss}$, was proposed, offering useful insights since it represents a trade off between maximum power gain ($P_{gain}\equiv P-P_{min}$) and minimum power loss ($P_{loss}\equiv P_{max}-P$), with respect to the minimum and maximum available power output for a heat engine. Besides, it is easy to implement in any energy converter, isothermal or non-isothermal, because it does not require the explicit evaluation of the entropy generation, and it is independent on environmental parameters.

Recently, it has been pointed out that the problem of local stability of operation regimes can be related with the regime’s optimization itself [20,21]. Thus, the effects of external perturbations on the operation regime and on the energetic properties such as power output, efficiency and entropy production, can be addressed. A natural question arises: Could entropy give us information to distinguish particular behaviors inside the operation regimes’ basin of attraction? Recent papers have explored this question using a somewhat simple model, the so-called low-dissipation heat engine (LD-HE), which departs from a first order approximation in the entropy generation of irreversible heat devices [5,22,23,24]. Despite its simplicity, this model can be related with more complex heat engines’ models [6,25,26,27,28,29,30,31,32,33,34]. These studies do not consider any specific information on the nature of the heat conduction mechanism but focus, instead, on dissipation and operation time symmetries, along with temporal constraints. Thus, due to its broad generality and independence of any heat transfer law, the low-dissipation approximation could be useful to provide general thermal properties for a variety of heat devices models in connection with its stability.

Among the mentioned connections of the low dissipation model, several scales are involved, from macroscopic models to microscopic ones. However, few systems have been analytically solved and limited precision in the control variables remains as an open question, being this the rationale for this stability study. In fact, in [35], it has been pointed out the lack of a systematic and quantitative description of how limited control affects the performance of heat engines. This problem is closely connected with some recent works regarding power fluctuations and large efficiencies (see, for example, [35,36,37]). In a related line of thought to this work, Pietzonka and Seifert proposed constancy as an additional ingredient in optimization [36].

Since the specific mechanisms that cause heat flows in LD-HE’s models are unknown, for the study of local stability, we introduce ad hoc restitutive forces to perform such stability analysis. In fact, different restitutive forces are associated with different system’s dynamics and stable points characteristics, as discussed in References [20,21]. This work focuses on the simplest case of restitutive forces, linearly dependent on deviations from the stationary state in power output and output heat flux released to the cold heat reservoir. Such forces yield a rich dynamics of the basin of attraction in terms of the symmetries and the external bath temperatures. Consequently, a relevant behavior on the entropy trajectories is observed in connection with the two optimized operation performance regimes.

The work is organized as follows. For self contained purposes, Section 2 summarizes some previous relevant results related with the LD-HE and the maximum power (MP) and maximum Omega (MΩ) operation regimes. In Section 3, the stability dynamics and the main characteristics of the basin of attraction in relation with irreversible and endoreversible behaviors are analyzed. In Section 4, a comparison of the evolution behavior of entropy production, power output and efficiency when the system evolves toward: (a) the stable point; or (b) the nullcline and eventually diverging to a nonphysical, no-heat engine state. Finally, some concluding remarks are presented in Section 5.

## 2. Low Dissipation Model and Maximum Power and Omega Regimes

The low-dissipation approximation considers a first order irreversible depart from a Carnot cycle. The dissipation occurs in the contact with thermal reservoirs. As usual, the adiabatic processes are consider as instantaneous. Accordingly, the exchanged heat with the cold (at temperature $T_{c}$) and hot (at temperature $T_{h}>T_{c}$) thermal reservoirs are modeled as [5]:

(1) $$\begin{array}{ccc} Q_{h} & = & T_{h}\Delta S\left(1-\frac{\Sigma_{h}}{\Delta St_{h}}\right), \end{array}$$

(2) $$\begin{array}{ccc} Q_{c} & = & T_{c}\Delta S\left(-1-\frac{\Sigma_{c}}{\Delta St_{c}}\right), \end{array}$$

where $t_{c}$, $t_{h}$ are, respectively, the contact times with the hot and cold reservoirs. ΔS is the entropy change of the baseline Carnot cycle along the hot isothermal process; and $\Sigma_{c}$, $\Sigma_{h}$ are the dissipation coefficients, containing all the information regarding intrinsic dissipation properties.

By defining the dimensionless variables ${\tilde{\Sigma }}_{c}\equiv \Sigma_{c}/\left(\Sigma_{c}+\Sigma_{h}\right)$, $\alpha \equiv t_{c}/\left(t_{c}+t_{h}\right)$ and $\tilde{t}\equiv \frac{\Delta S}{\Sigma_{T}}\left(t_{c}+t_{h}\right)$, being $\Sigma_{T}\equiv \Sigma_{c}+\Sigma_{h}$, the main thermodynamic functions, total entropy production (${\dot{\Delta S}}_{tot}$), efficiency and power output, can be written dimensionless as follows [23]:

(3) $${\dot{\tilde{\Delta S}}}_{tot}=\frac{1}{{\tilde{t}}^{2}}\left(\frac{1-{\tilde{\Sigma }}_{c}}{1-\alpha }+\frac{{\tilde{\Sigma }}_{c}}{\alpha }\right),$$

(4) $$\tilde{\eta }=\eta =1-\tau \frac{1+\frac{{\tilde{\Sigma }}_{c}}{\alpha \;\tilde{t}}}{1-\frac{1-{\tilde{\Sigma }}_{c}}{\left(1-\alpha \right)\tilde{t}}}$$

(5) $$\tilde{P}=\frac{1}{\tau \;\tilde{t}}\left(1-\tau -\frac{1-{\tilde{\Sigma }}_{c}}{\left(1-\alpha \right)\tilde{t}}-\frac{\tau \;{\tilde{\Sigma }}_{c}}{\alpha \;\tilde{t}}\right).$$

where ∼ refers to energy weighted by the heat output from the baseline Carnot cycle, $T_{c}\Delta S$; and the currents are obtained by dividing by the dimensionless total time $\tilde{t}$. According to the above stated definition, the Ω function is given by $\Omega =2P-P_{min}-P_{max}=2\left(2\eta -\eta_{C}\right)Q_{h}$, whose dimensionless form is

(6) $$\tilde{\Omega }=\frac{1-\tau }{\tau \;\tilde{t}}-\frac{\left(1+\tau \right)\left(1-{\tilde{\Sigma }}_{c}\right)}{\left(1-\alpha \right){\tilde{t}}^{2}}-\frac{2{\tilde{\Sigma }}_{c}}{\alpha \;{\tilde{t}}^{2}}.$$

The main results related to power output and Omega function maximization are presented.

In the LD-HE model τ and ${\tilde{\Sigma }}_{c}$ do not play a role in the optimization, since ${\tilde{\Sigma }}_{c}$ involves intrinsic properties of the device. For τ and ${\tilde{\Sigma }}_{c}$ given, both MP and MΩ operation regimes accept global optimization through α and $\tilde{t}$.

The values that maximize $\tilde{P}$ and $\tilde{\Omega }$ are [24]

(7) $$\begin{array}{ccc} \alpha^{*} & = & \left\{\begin{array}{cc} \frac{1}{1+\sqrt{\frac{1-{\tilde{\Sigma }}_{c}}{\tau {\tilde{\Sigma }}_{c}}}} & MP,\\ \frac{1}{1+\sqrt{\frac{\left(1+\tau \right)\left(1-{\tilde{\Sigma }}_{c}\right)}{2\tau {\tilde{\Sigma }}_{c}}}} & M\Omega , \end{array}\right. \end{array}$$

(8) $$\begin{array}{ccc} {\tilde{t}}^{*} & = & \left\{\begin{array}{cc} \frac{2}{1-\tau }{\left(\sqrt{\tau {\tilde{\Sigma }}_{c}}+\sqrt{1-{\tilde{\Sigma }}_{c}}\right)}^{2} & MP,\\ \frac{2}{1-\tau }{\left(\sqrt{2\tau {\tilde{\Sigma }}_{c}}+\sqrt{\left(1-{\tilde{\Sigma }}_{c}\right)\left(1+\tau \right)}\right)}^{2} & M\Omega . \end{array}\right. \end{array}$$

From now on, * indicates the steady-state value at MP or MΩ conditions. As expected, $\eta_{M\Omega }\geq \eta_{MP}$ is obtained. Depending on the dissipation coefficient asymmetries, ${\tilde{\Sigma }}_{c}$, both efficiencies take values on the following intervals:

(9) $$\begin{array}{c} \frac{\eta_{C}}{2}\leq \eta_{MP}\leq \frac{\eta_{C}}{2-\eta_{C}}, \end{array}$$

(10) $$\begin{array}{c} \frac{3\eta_{C}}{4}\leq \eta_{M\Omega }\leq \frac{3-2\eta_{C}}{4-3\eta_{C}}\eta_{C}, \end{array}$$

where $\eta_{C}=1-T_{c}/T_{h}$ is the Carnot efficiency. The upper bounds are achieved in the limit ${\tilde{\Sigma }}_{c}\to 0$ and the lower bounds when ${\tilde{\Sigma }}_{c}\to 1$. In the symmetric case (${\tilde{\Sigma }}_{c}=1/2$), both regimes efficiencies are:

(11) $$\begin{array}{c} \eta_{MP}^{sym}=1-\sqrt{1-\eta_{C}}=1-\sqrt{\tau }=\eta_{CA}, \end{array}$$

(12) $$\begin{array}{c} \eta_{M\Omega }^{sym}=1-\sqrt{\frac{\left(1-\eta_{C}\right)\left(2-\eta_{C}\right)}{2}}=1-\sqrt{\frac{\tau \left(1+\tau \right)}{2}}, \end{array}$$

where the paradigmatic Curzon–Ahlborn efficiency, $\eta_{CA}=1-\sqrt{T_{c}/T_{h}}$, is obtained for the MP case. On the other hand, efficiency and entropy production only accept optimization through α. For this reason, the MΩ figure of merit represents a suitable choice to compare the dynamics toward relaxation of the MP regime with another less entropy producing regime.

## 3. Local Stability

As proposed in [20], the optimization variables α and $\tilde{t}$ are dynamic variables governed via a dynamic system that considers departures from the stationary state (operation regime). The restitution forces are model as functions of ${\dot{\tilde{Q}}}_{c}$ and $\tilde{P}$. The currents associated to α and $\tilde{t}$ are [20]:

(13) $$\begin{array}{cc} \frac{d\alpha }{dt} & =f\left({\dot{\tilde{Q}}}_{c}\left(\alpha ,\tilde{t}\right)\right), \end{array}$$

(14) $$\begin{array}{cc} \frac{d\tilde{t}}{dt} & =g\left(\dot{\tilde{P}}\left(\alpha ,\tilde{t}\right)\right). \end{array}$$

To guarantee stability, *f* and *g* must be monotonically decreasing functions fulfilling that in the stationary state $f\left({\tilde{Q}}_{c}\left(\alpha^{*},{\tilde{t}}^{*}\right)\right)=g\left(\tilde{P}\left(\alpha^{*},{\tilde{t}}^{*}\right)\right)=0$. The simplest way to guarantee this [38,39,40] is by assuming that the dynamics is described by the following linear ODE system with respect to ${\dot{\tilde{Q}}}_{c}$ and $\tilde{P}$:

(15) $$\begin{array}{ccc} \frac{d\alpha }{dt} & = & C\left({\dot{\tilde{Q}}}_{c}\left(\alpha^{*},{\tilde{t}}^{*}\right)-{\dot{\tilde{Q}}}_{c}\left(\alpha ,\tilde{t}\right)\right), \end{array}$$

(16) $$\begin{array}{ccc} \frac{d\tilde{t}}{dt} & = & D\left(\tilde{P}\left(\alpha^{*},{\tilde{t}}^{*}\right)-\tilde{P}\left(\alpha ,\tilde{t}\right)\right), \end{array}$$

where *C* and *D* are positive constants and give the strength of the restitution forces, the larger they are, the fastest the system will evolve.

**Figure 1:**
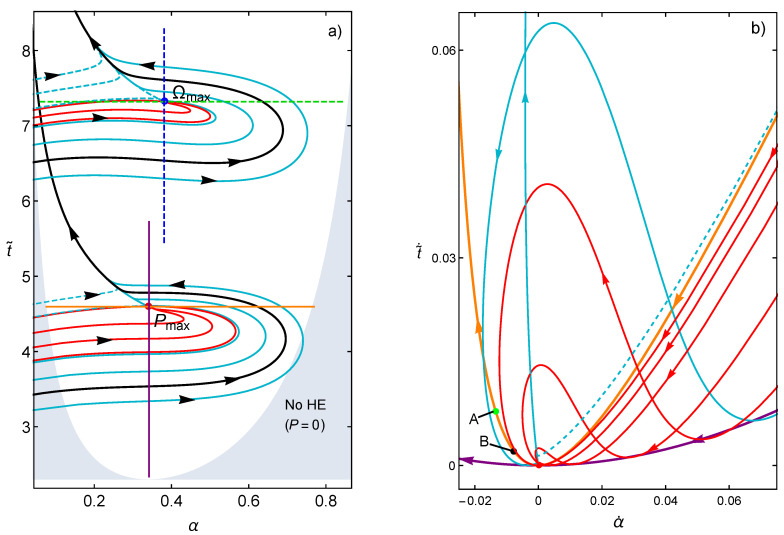
(**a**) Computed trajectories stemming from solving Equations (15) and (16) around the MP and MΩ regimes. C=D=1 and ${\tilde{\Sigma }}_{c}=\tau =2/5$ are used. The No HE label refers to the region where P≤0, represented by the shaded region. Blue continuous trajectories surround the basin of attraction and arrive to the nullcline; a representative case of this is shown in black. Dashed blue curves start at larger times and arrive to the nullcline from the left. Red lines are inside the basin of stability. The constraints α=constant and $\tilde{t}=constant$ are depicted in both regimes. They produce loop-like and open curves in the *P*–η plane, respectively, which are typical for irreversible and endoreversible heat engines, respectively. (**b**) The corresponding $\dot{\alpha }$ vs. $\dot{\tilde{t}}$ curves are depicted. The irreversible and endoreversible limits are shown. The labels *A* and *B* on the irreversible limit correspond to the points of minimum entropy production and maximum efficiency, respectively (see text for a more detailed explanation). Arrows denote the evolution direction.

In Figure 1, representative trajectories around the stationary state are plotted by solving Equations (15) and (16). The main feature is the existence of a well defined basin of attraction with a rich dynamics around the stable point. In Figure 1, three kind of curves are explicitly denoted. The red curves evolve to the stationary state while the rest of the curves evolve to the nullcline where $\frac{d\alpha }{dt}=0$, either from the left (dashed blue curves) or from the right (solid blue curves). A black curve is shown as a representative case of a trajectory that surrounds the stability basin. The thermodynamics behavior in the three kind of trajectories are by no means obvious, and the influence of time constraints, as shown below, could indeed have relevant information on this regard.

In Figure 1, vertical and horizontal lines for constant values of α or $\tilde{t}$, respectively, are also explicitly labeled in both cases. In Reference [41], it is shown that these time constraints reproduce open and closed behaviors for the power vs. efficiency curves, typical from endoreversible (all irreversibilities coming from the coupling to external heat thermal baths) and irreversible (including irreversibilities coming from heat leaks and internal dissipation) heat engines. In Figure 1b, the evolution of the same trajectories is shown in the $\dot{\alpha }$ vs. $\dot{\tilde{t}}$ plane. The endoreversible limit comprise the points where for the same velocity in α, one obtains the smaller velocity in the operation time $\tilde{t}$ (which is related to the irreversibility of the system). Notice how, as the system evolves, the trajectories tend to reach the endoreversible limit first, after that those evolving in the basin of attraction (red color) enter into a dynamics bounded by the irreversible limit while some trajectories arriving as the nullcline (blue color) can cross this limit.

The influence of the irreversible and endoreversible limits on the dynamic evolution of the system are further addressed in the next section, which contains a thermodynamic analysis of the relaxation trajectories to find the characteristic energetic behaviors associated with the basin of attraction.

## 4. Entropy, Efficiency and Power Evolution Toward Relaxation

The role of entropy and internal energy as stability criteria in thermodynamics is well known. In what appears a separate subject, the goal of optimization of energy converter is focused in obtaining the larger efficiencies and power-output with the less entropy generation, with the simultaneous optimization of the three quantities being an impossible task. In this section, we show that stability favors, to some extent, the simultaneous optimization of the three thermodynamics functions.

**Figure 2:**
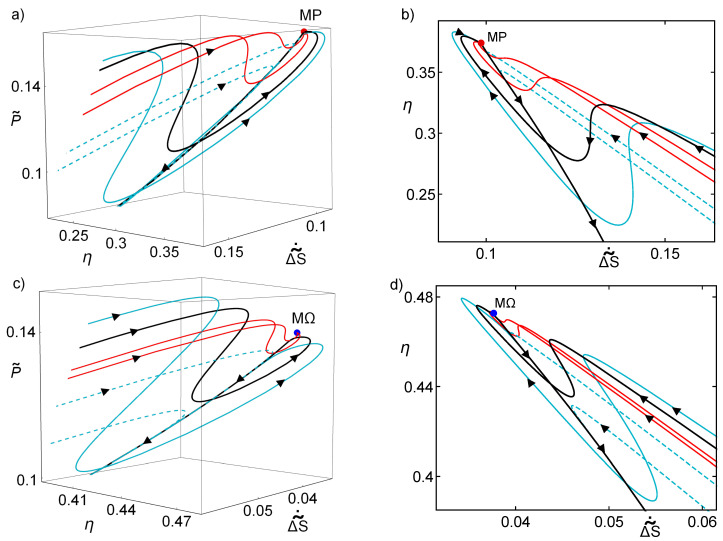
By solving Equations (15) and (16), we obtained trajectories on the $\left(\eta ,\tilde{P},\dot{\tilde{\Delta S}}\right)$ space. The depicted trajectories stem from the parameterization appearing in Figure 1. The color and line-type codes are the same to distinguish different behaviors from the phase space. (**a**) The case of MP regime; (**b**) the same trajectories are shown but emphasizing the relation between entropy production and efficiency; (**c**) the MΩ case is shown; and (**d**) the corresponding figure highlighting the entropy production vs. efficiency. In all cases, the trajectories evolve toward the cusp in each surface. $\tau ={\tilde{\Sigma }}_{c}=0.4$ are used.

By solving numerically Equations (15) and (16), the trajectories $\left(\alpha \left(t\right),\;\tilde{t}\left(t\right)\right)$ can be computed and, by substituting them in Equations (3)–(5), the evolution of $\tilde{\Delta S}$, η and $\tilde{P}$ in the relaxation after a perturbation on the system is obtained. These trajectories are depicted in Figure 2 over the η–$\tilde{P}$–$\dot{\tilde{\Delta S}}$ surface. Figure 2a,b shows some of these trajectories around the MP state and Figure 2c,d shows some trajectories around the MΩ state. The difference between Figure 2a,b is only the view point, the latter making emphasis in the relation between entropy and efficiency. The same is done for Figure 2c,d for the MΩ regime. As in Figure 1, blue continuous curves are those that go around the basin of attraction and arrive to the nullcline $\frac{d\alpha }{dt}=0$, the black curve represents one of them, that orbits close to the stable point but do not converge to it. The dashed trajectories involves perturbation where $\tilde{t}>{\tilde{t}}_{MP}$ and arrive to the nullcline from the opposite side. Red trajectories are inside the basin of attraction for the MP state and they converge to the stationary state.

The dynamics around the MP stable point is such that, far from it, the solid blue curves evolve toward the upper values of $\tilde{P}$ in trajectories of increasing efficiency and decreasing entropy (more noticeable in Figure 2b). The curves start to orbit states of larger power while moving to the true MP state. In these oscillations, there are drops of power and efficiency and an increment of entropy. After one last oscillation that misses the stable point, the curves decay toward the nullcline in a trajectory where power output and efficiency decrease, and entropy production increases, finally ending in the no-HE region where the power output and efficiency are negative.

On the other hand, the dashed blue curves are such that they enter directly into the nullcline. The trajectories inside the basin of attraction follow the same dynamic as the blue curves but they present one small oscillation with a small power and efficiency drop and they rather enter into an orbit that quickly drives them to the stable point. These small drops of power and efficiency and very small increment of entropy, which only occurs close to the stable point, could be indeed the true difference between the states inside the basin of attraction and those states that evolve toward the nullcline.

The dynamics on the MΩ regime follows a very similar pattern, as depicted in Figure 2c,d. The evolution of the curves still present oscillations over values of the power output, even when the fixed point is not the MP. A distinctive feature is that the trajectories are more lined and with a narrower basin of attraction. Notice that, when comparing with the MP results, the MΩ regime implies smaller entropy production trajectories and higher efficiency, in agreement with the bounds accounted for Equations (9) and (10) for high asymmetries and Equations (11) and (12) for the symmetric case. Notice in Figure 2c,d the different scales in entropy and efficiency with respect to Figure 2a,b

Therefore, all the above provides a firm basis to consider that stability could be linked to a compromise of performance among η, $\tilde{P}$, $\dot{\tilde{\Delta S}}$, in what we can call a thermodynamic “self-improvement” in the process of relaxation, with the smallest possible fluctuations on the performance of the engine.

**Figure 3:**
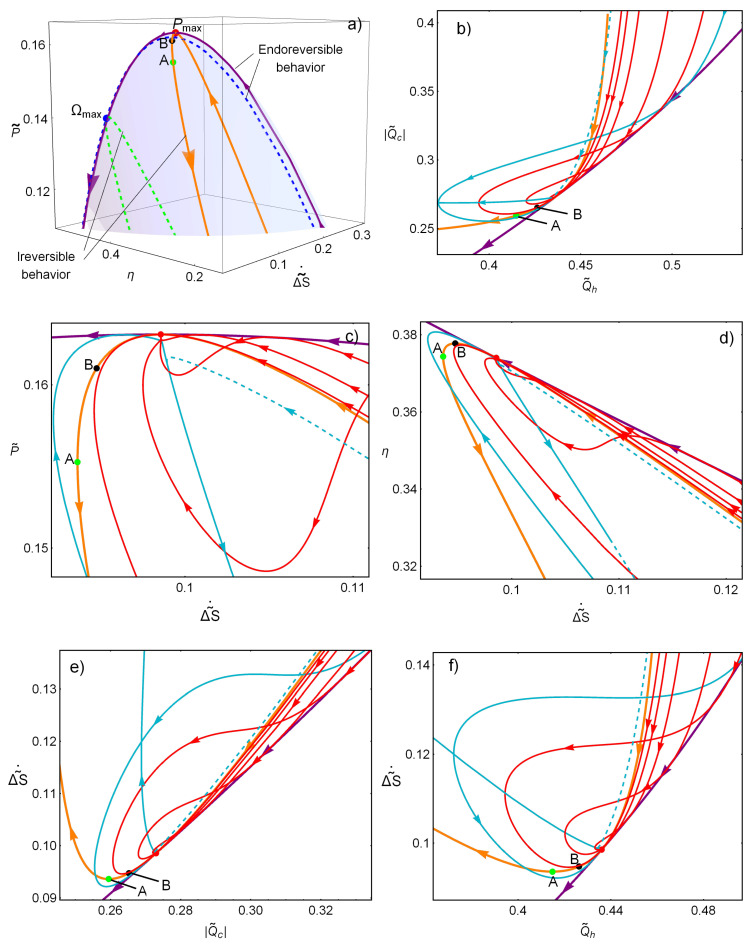
(**a**) The resulting endoreversible and irreversible behaviors, produced by the time constraints α=constant and $\tilde{t}=constant$, respectively, are depicted in a *P*–η–$\dot{\tilde{\Delta S}}$ surface; in continuous lines for MP and in dashed lines for MΩ. (**b**) ${\dot{\tilde{Q}}}_{h}$ vs. $-{\dot{\tilde{Q}}}_{c}$ is shown. (**c**,**d**) The power and efficiency evolution, respectively, as a function of the entropy generation. (**e**,**f**) The evolution of entropy production as the output and input heat vary due to the relaxation dynamics. (**b**–**f**) The endoreversible limit clearly offers a better compromise in the use of energy. $\tau ={\tilde{\Sigma }}_{c}=0.4$ are used. (**b**–**f**) Plots for the MP regime.

An interesting feature results from comparing the relaxation trajectories with the open (endoreverversible) and closed (irreversible) behaviors depicted in Figure 3a, where the irreversible and endoreversible limits are displayed over the surface $\tilde{P}$-$\dot{\tilde{\Delta S}}$-η, in continuous lines for the MP state and in dashed lines for the MΩ state. Both curves cross at the MP state, however, the irreversible limit displays, additionally, points of minimum entropy production (labeled as *A*) and maximum efficiency (labeled as *B*). The orientation of the arrows indicate the direction at which either α or $\tilde{t}$ increase (from 0 to 1, and from 1 to *∞*, respectively). It can be noticed that these times constraints are not arbitrarily located with respect to the trajectories on the basin of attraction.

As explained above, regarding Figure 1b, far from the stable point, all trajectories tend to reach the endoreversible limit (given by the constraint $\alpha =\alpha_{P_{max}}$), while, near the stationary state, the trajectories are bounded by the irreversible limit given by $\tilde{t}={\tilde{t}}_{P_{max}}$. The reason for this is a hierarchy/distinction in the relaxation behavior due to a thermodynamic self improvement attached to the stability dynamics. In the linear approximation (near the stationary state), it was obtained that the stability depends only on the relaxation of α [24], corresponding to the time constraint depicted in Figure 1a (horizontal line with $\tilde{t}={\tilde{t}}_{P_{max}}$). Thus, trajectories in the basin of attraction are bounded by the irreversible limit; on the other hand, far from the stationary state, the trajectories display a preference for the endoreversible limit, which offers a number of advantageous features.

The preference to evolve toward the endoreversible limit can be understood by looking into Figure 3b–f which apply for the MP regime (although the results are also valid for the MΩ state). In Figure 3b, the endoreversible limit denote the lower value of heat waste for any given heat input. In Figure 3c,d, the endoreversible limit gives the larger power and efficiency for a given entropy production. Figure 3e,f shows that, additionally, it gives the lower entropy production for given heat output and input, respectively. Thus, the system evolution reveals a preference for a better compromise displayed by the endoreversible configuration, and the time constraints influence in a major way the dynamics toward relaxation.

**Figure 4:**
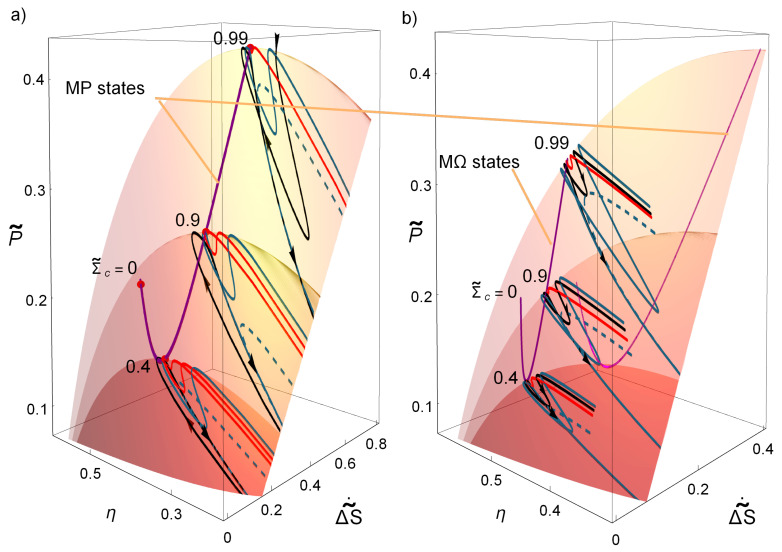
Dynamics described by Equations (15) and (16): local-stability of the MP state (**a**); and the MΩ state (**b**) over the surfaces $\left(\eta ,\tilde{P},\dot{\tilde{\Delta S}}\right)$ for three cases of ${\tilde{\Sigma }}_{c}=\left\{0.4,0.9,0.99\right\}$ with τ=0.4. The surfaces with larger ${\tilde{\Sigma }}_{c}$’s contains those with smaller values of ${\tilde{\Sigma }}_{c}$. Over these surfaces lies the curve of all MP states for ${\tilde{\Sigma }}_{c}\in \left(0,1\right)$. In all cases, the trajectories evolve with oscillations over the cuspid.

Next, the influence of the asymmetries keeping a fixed thermal gradient it is presented. Figure 4 shows the η, $\tilde{P}$, $\dot{\tilde{\Delta S}}$ surfaces for three cases of ${\tilde{\Sigma }}_{c}=\left\{0.4,0.9,0.99\right\}$ and for all possible values of $\alpha \in \left(0,1\right)$ and $\tilde{t}\in \left(0,50\right)$; in Figure 4a for MP and in Figure 4b for MΩ. Over the depicted surfaces lies the curve of MP states for all $\Sigma_{c}\in \left(0,1\right)$ (purple curve). The three MP states ${\tilde{\Sigma }}_{c}=\left\{0.4,0.9,0.99\right\}$ represented in the figure have their own dynamics toward the stable point. Some representative trajectories are displayed over each surface. In this figure, it is noticeable that relaxation trajectories are more likely to evolve in the direction of increasing power and efficiency and decreasing entropy. Those arriving to the stable point, as stated above, have the smallest variations in entropy and efficiency, while all the trajectories arriving to the nullcline evolve to states of less power, less efficiency and larger entropy production. Thus, systematically in the search for the true MP, the orbits around the stable point always search for more “convenient” thermodynamic states. It can be also seen in Figure 4 how larger values of ${\tilde{\Sigma }}_{c}$ produce narrower basins of attraction, thus yielding trajectories with less variations of power output, efficiency and entropy production. The MΩ states, in comparison with MP states, have a better energetic evolution (less drops) but with the cost of having more constrained states.

**Figure 5:**
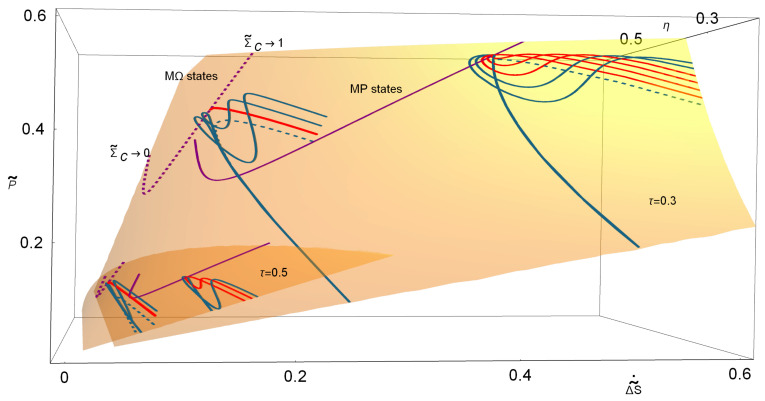
Relaxation evolution for (τ={0.3,0.5}) and for the two operation regimes. The curves of all MP and MΩ states for ${\tilde{\Sigma }}_{c}\in \left(0,1\right)$ are shown. C=D=1 and ${\tilde{\Sigma }}_{c}=0.9$ are used.

Finally, Figure 5 shows the difference on the dynamics when different thermal gradients are considered. As τ decreases the surface where the evolution is held diminish its area and the trajectories are more leaned. As the thermal gradient is larger, the basin of attraction accepts larger perturbations but the nature of the trajectories is the same.

## 5. Conclusions

The analysis of the dynamics behind local stability of operation regimes, such as maximum power and maximum Ω functions, reveals a feature that recently has started to gain certain attention: stability/constancy of heat devices should be considered as an additional ingredient in the optimization of heat devices; and there exists a kind of trade-off among stability, power output and efficiency. Here, the entropy production is added as another relevant aspect to consider.

It has been found that, for irreversible processes obeying the small dissipation assumption, the stability basin is associated with thermal behaviors of simultaneous improvement of the main thermodynamics quantities, power, efficiency and entropy, as well as small drops of their performance. A comparison of the relaxation trajectories with the open and closed *P* vs. η behaviors of endoreversible and irreversible heat engines reveals that, far from the stable point, the trajectories tend to evolve to the endoreversible limit case, while, close to the stable point, the evolution occurs inside the region delimited by the irreversible limit behavior, and all trajectories crossing this boundary will diverge to a non-physical state. These qualitative behaviors apply for every value of the dissipation coefficient ${\tilde{\Sigma }}_{c}$ and for any thermal gradient, given by τ, although small numerical differences can be observed for concrete numerical values of these parameters.
